# Engaging With Health Consumers in Scientific Conferences—As Partners not Bystanders

**DOI:** 10.1111/hex.14147

**Published:** 2024-07-17

**Authors:** Bronwyn Newman, Janelle Bowden, Rebecca Jessup, Lauren J. Christie, Ann Livingstone, Mitchell Sarkies, Anagha Killedar, Carole Vleeskens, Mashreka Sarwar, Thit Tieu, Saran Chamberlain, Reema Harrison, Alison Pearce

**Affiliations:** ^1^ Australian Institute of Health Innovation Macquarie University Sydney New South Wales Australia; ^2^ AccessCR Pty Ltd Sydney New South Wales Australia; ^3^ Staying Well Programs Northern Health Melbourne Victoria Australia; ^4^ School of Allied Health, Human Services and Sport La Trobe University Bundoora Victoria Australia; ^5^ Allied Health Research Unit St Vincent's Health Network Sydney Darlinghurst New South Wales Australia; ^6^ Faculty of Health Sciences Australian Catholic University North Sydney New South Wales Australia; ^7^ St Vincent's Health Network Sydney Nursing Research Institute, St Vincent's Hospital Melbourne and Australian Catholic University Fitzroy Victoria Australia; ^8^ Deakin Health Economics, School of Health and Social Development, Institute for Health Transformation, Faculty of Health Deakin University Geelong Victoria Australia; ^9^ Sydney School of Health Sciences, Faculty of Medicine & Health University of Sydney Camperdown New South Wales Australia; ^10^ Sydney Health Partners, Implementation Science Academy University of Sydney Camperdown New South Wales Australia; ^11^ Menzies Centre for Health Policy and Economics, School of Public Health, Faculty of Medicine and Health The University of Sydney Sydney New South Wales Australia; ^12^ Consumer Presenter, HSRAANZ Conference; ^13^ Consumer Research Partner, Consumer PCC Initiative (Channelling Consumer Voices To Transform Person‐Centred Care); ^14^ SPHERE Musculoskeletal Clinical Academic Group Consumer Community Council Sydney New South Wales Australia; ^15^ UniSA Allied Health & Human Performance University of South Australia Adelaide South Australia Australia; ^16^ School of Public Health The University of Sydney Sydney New South Wales Australia

**Keywords:** collaboration, conferences, consumer engagament, research dissemination

## Abstract

**Introduction:**

It is now widely recognised that engaging consumers in research activities can enhance the quality, equity and relevance of the research. Much of the commentary about consumer engagement in research focuses on research processes and implementation, rather than dissemination in conference settings. This article offers reflections and learnings from consumers, researchers and conference organisers on the 12th Health Services Research Conference, a biennial conference hosted by the Health Services Research Association of Australia and New Zealand (HSRAANZ).

**Method:**

We were awarded funds via a competitive application process by Bellberry Limited, a national not‐for‐profit agency with a focus on improving research quality, to incorporate consumer engagement strategies in conference processes and evaluate their impact.

**Findings:**

Strategies included consumer scholarships, a buddy system, designated quiet space and consumer session co‐chairs; the reflections explored in this paper were collected in the funded, independent evaluation. Our insights suggest a need for more structured consumer involvement in conference planning and design, as well as the development of specific engagement strategies.

**Conclusion:**

To move toward active partnership in scientific conference settings, our experience reinforces the need to engage consumers as members in designing and conducting research and in presenting research and planning conference content and processes.

**Public Contribution:**

Consumer engagement in research dissemination at conferences is the focus of this viewpoint article. Consumers were involved in the conception of this article and have contributed to authorship at all stages of revisions and edits.

## 1 Introduction/Background

It is now well recognised that engaging with consumers in designing and conducting research enhances the benefits and relevance of research to end users [1]. Academic publication about collaborations between consumers and researchers has rapidly expanded alongside the increasing engagement of consumers in research [1]. However, much of this literature focuses on the process, benefits and considerations when working together and implementation of research into policy and practice [2]. ‘Consumer engagement’ in research should be considered across a spectrum of activity from informing, consulting through to partnership or consumer‐led activities [3]. The role of collaboration between researchers and consumers in disseminating research findings has received limited attention [4]. Scientific conferences are a major avenue for collaboration and research dissemination. The value of health consumer participation and involvement in these events has been demonstrated; however, little has been published on how best to support consumer participation [5]. This article offers reflections and learnings from the involvement of consumers in the 12th Health Services Research Conference, a biennial conference hosted by the Health Services Research Association of Australia and New Zealand (HSRAANZ). The authors comprise a diverse group of consumers, researchers and conference organisers across different career stages.

The HSRAANZ is the key professional association for individuals and groups in Australia and Aotearoa, New Zealand, undertaking, implementing and disseminating health services and health systems research. HSRAANZ's purpose is to ‘facilitate communication across researchers, and between researchers and policymakers, promote education and training in health services research, and ensure sustainable capacity in health services research in Australia and New Zealand’ [6]. The biennial Health Services Research Conference is the premier scientific meeting for researchers, policymakers and practitioners using health services research to improve health service delivery and health outcomes. In 2022, the conference attracted 420 registered delegates, including nine consumers and delegates from a range of disciplines. The 2022 conference was intentionally planned to reflect this commitment and to promote a consumer‐centric lens to health services research in Australia and Aotearoa, New Zealand.

The term ‘health service consumer’ or ‘consumer’ describes ‘people who use health services, as well as their family and carers’ [7]. While widely used in the Australian context, the term ‘consumer’ is not always preferred; alternatives like ‘person with lived experience’, ‘advocate’ or ‘lived experience researcher’ are also used by consumers to identify their role.

Health service consumers were actively invited to participate in the Health Services Research Conference in 2022, as both delegates and presenters. Activities to enhance consumer engagement in the conference were included based on evidence, suggesting that such activities increase research dissemination and offer a venue for conversations about research relevant to consumers [5]. Of the nine consumers who attended the conference, six co‐chaired sessions and four consumers presented papers. Bellberry Limited (https://bellberry.com.au/), a national private not‐for‐profit organisation with a focus on improving research quality, provided funding to support a consumer‐centred conference programme. The funding supported a consumer keynote presentation, a session focused on consumer engagement in research, a workshop on meaningful consumer engagement, five consumer scholarships for conference attendance and a process evaluation.

Reflections are provided on findings from the evaluation, which obtained feedback and insights from conference attendees, presenters and conference organisers. Participants chose to contribute to the evaluation voluntarily; this was not a condition of accepting a scholarship and all data were deidentified before sharing with conference organisers. The evaluation was conducted by an independent evaluator using attendance at and observations of conference proceedings, along with voluntary face‐to‐face or telephone interviews with nine consumers and 10 researcher delegates conducted at the conference or within 2 weeks. While not exhaustive, this commentary aims to share experiences and provide insights into further opportunities for consumers to partner in disseminating research findings in conference settings. Reflections are provided in two parts: 1. discussion of the practical strategies employed and 2. consideration of the concept of pursuing partnership between consumers and researchers in research dissemination more broadly.

1. Practicalities, strategies and reflections

**Figure 1:**
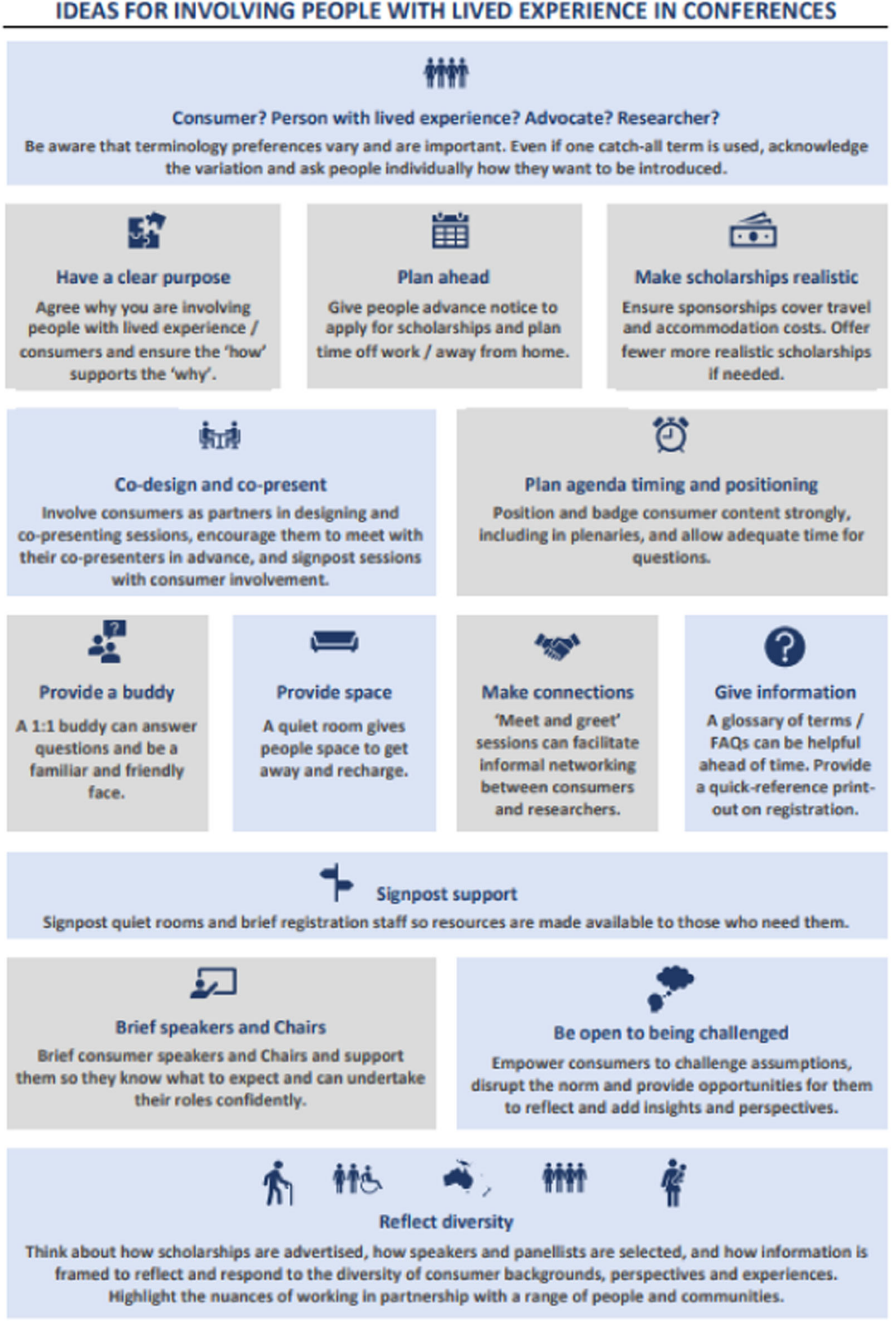
Ideas for involving people with lived experience in conferences. *Increasing consumer engagement in the Health Services Research Conference 2022*, Infographic from Summary Evaluation Report 10 February 2023 Prepared by Alison Evans Consulting.

To foster consumer engagement at the conference, we collaborated with three consumer partner organisations in planning, implementing and promoting our approach. The three agencies (Access CR, Health Consumers NSW and Cancer Voices NSW) have varied roles in supporting healthcare consumers, in advocacy, access to care and research participation. Our approach was underpinned by the four principles of inclusive research: equality, reciprocity, accessibility and diversity [8]. Aligned with these principles, we collaboratively developed the following strategies; an infographic summary is provided in Figure 1.

1.1. Scholarships were offered to address issues of equity of opportunity to attend the conference by supporting consumers financially. The scholarships were reviewed by a panel that looked for diverse representation (across ethnicity, gender and lived experience with health conditions) of consumer participants. These scholarships covered the registration fee and a contribution towards travel and accommodation costs. Scholarships were advertised via the HRSAANZ membership and broader mailing list, social media networks and through the three partnering consumer organisations. Sixteen applications were received, with five scholarships awarded: four supporting in‐person attendance and one for the ‘on demand’ conference platform. Successful recipients valued the straightforward application process. The total number of scholarships was limited by the funding available. A greater number of scholarships in relation to the overall number of attendees may reduce the demand on individual consumer attendees.

Consumer feedback suggested that advertising the scholarship opportunity and confirming successful applicants at least 2–3 months before the conference would be useful, particularly for rural attendees and carers. Ensuring that the costs for participants, including people living in rural and remote areas, are fully covered was suggested along with the need for wider dissemination of scholarship information to enhance the diversity of participants across varied socioeconomic, geographic, culturally and linguistically diverse groups.

1.2. To enhance accessibility and reciprocity opportunities to build connections, opportunities for genuine connections were offered throughout the conference. These included the following:

1.2.1. A buddy system to connect consumers and researchers. Members of the conference organising committee nominated themselves as buddies, and invited researcher registrants were invited to participate via an expression of interest. All consumer attendees were offered the opportunity to be paired with a researcher buddy. Consumer and researcher buddies were paired after discussion and consensus within the committee considering the previous experience of both buddies and researchers. Initial contact between buddies was encouraged by meeting face to face at the allocated consumer quiet space during the first break on Day 1 of the conference. Consumers reported that they engaged in different ways with buddies; some indicated that they plan to have ongoing contact after the conference to engage in further research activities and others had minimal contact throughout the conference event. For future events, consideration will be given regarding the accessibility of conference promotion, both in content and in the avenues selected to promote the conference. Ideally, the glossary and conference information will be provided several weeks or months ahead of the event, as suggested in the evaluation.
1.2.2. Consumer co‐chairs were a feature of the consumer‐focused day of the conference. Consumers co‐chaired six sessions to enhance opportunities for reciprocity and diversity. Researchers and consumers appreciated clear guidance on the chairing role and opportunities to prepare and interact with co‐chairs before the session. Some delegates (both consumer and researcher) reported that more guidance for the researcher chairs would have enabled them to work more collaboratively with consumer co‐chairs, particularly to support them when fielding questions.
1.2.3. Optional consumer lanyards were provided to enable consumer attendees to be easily identified for conversations and networking. Feedback about this strategy was mixed. Consumers and researchers noted a need for greater consultation with this approach to determine the value that this form of identification adds for both consumer and researcher attendees.

1.3. Conference content from research utilising participatory approaches was actively encouraged in invitations for abstract submissions. The conference programme deliberately prioritised opportunities for consumers to present and share research, as well as offering focused sessions and a workshop for researchers on consumer engagement. Four consumers presented at the conference.

Researchers, health service staff and consumers reported that they primarily attended the conference to learn more about health service research and network with fellow attendees. However, consumers identified that they were particularly motivated by the opportunity to engage in advocacy (viewed by many health consumers as one of their primary roles) to drive meaningful change and this impacted the perceived relevance of some presentations. Some consumers reported being disappointed that the research presentations were technical rather than pitched appropriately for community or consumer audience members. For future conferences, presenters should receive instruction regarding the anticipated audience and ‘pitch’ their presentation to make the content more accessible for all attendees.

Clear, transparent information to describe the focus and intent of the conference as well as the role and expected benefits of attendance to consumers, was identified as a useful strategy for future conferences to align participant expectations. Delegates suggested creating specific streams with a consumer focus, grouping the most relevant presentations for consumers or indicating in the programme which sessions are designed to appeal to both consumer and researcher audiences.

1.4. Practical strategies to support participation were implemented at various stages during the conference. For example, chairs and speakers were briefed about avoiding jargon and using inclusive language and consumers were provided with definitions of key health service research terms or acronyms via a glossary. The event was physically accessible and inclusive; for example, the venue was wheelchair‐accessible, equipped with hearing loops, quiet spaces and a parents' room.

An allocated quiet space was created at the conference venue for consumer conference delegates to meet and reflect between sessions. One consumer noted the need for clarity in the use of space; for example, the quiet area should not be used for online meetings or telephone conversations for conference attendees. Future conference organisers could also canvas cultural and spiritual needs, for example, offering a prayer room. For future events, consideration will be given regarding the accessibility of conference promotion, both in content and in the avenues selected to promote the conference. Ideally, the glossary and conference information will be provided several weeks or months ahead of the event, as suggested in the evaluation.

The strategies described above fostered engagement with consumers in the Health Services Research Conference, and the evaluation highlighted the potential for further enhancement. Consumer and researcher delegates reinforced the value of consumer perspectives throughout the conference planning process. Evaluation participants reinforced the need for consumer representation in the planning committee, as well as in development of strategies implemented to engage consumers. Consumers also highlighted the need for engagement in planning conference content, particularly the importance of a conference programme reflective of consumer priorities, preferences and motivations for attendance.

This evaluation indicated that there were many individual and collective benefits of including consumers in the Health Services Research Conference. During the conference, almost all researcher conference delegates reported engaging with consumers a little or a lot and reported that it was an important component for future conferences. There were several anecdotes of significant connections forged between consumer and researcher delegates, some individual interactions leading to further opportunities for research collaboration (e.g., a consumer investigator on a Medical Research Future Fund research project), with other buddies reporting that they plan to remain in contact to explore future opportunities. Beyond these benefits to individuals, delegates reported that the conference was collectively enhanced by including consumer perspectives and the richness of diverse experiences.

2. Pursuing partnership or consumers as ‘partners not bystanders’

The desire to move from consumers as ‘bystanders’ to partners in research conferences was a strong message from the evaluation. Consumers have distinct perspectives to bring to research conferences with diverse experiences as end users of health services, co‐researchers and as advocates or ‘change agents’. Moving from the traditional researcher/practitioner focus of health service conferences to an approach more inclusive of consumers as partners requires a significant transition in mindset and conference processes, reflective of the broader shift within the health system and research activities [3]. The strategies described are a useful starting point for including consumers in conference processes and proceedings. These findings highlight that considerations are needed to make conferences more inclusive of consumer participants and operationally, this takes foresight, time, structure and funding. Increasing opportunities for consumers to shape the topics and approaches illuminates the present challenge to move from inclusive strategies to collaborative partnerships.

Open, transparent discussion between researchers and consumers for research dissemination is a feature of respectful research collaboration that includes and values consumer perspectives [3]. Consumer engagement in conferences to enrich research dissemination is valued by researchers; however, further exploration is needed to confirm the value of conferences to consumers. Given the low number of scholarship applications, it would be useful to explore consumer perceptions of conferences and motivations for/value placed on attending them. Through the evaluation, consumers reflected that they attend health service conferences for an array of reasons, highlighting the need to consider motivations for attendance when planning content and options. Consumers saw their perspective as aligned, yet distinct from researchers or professional delegates. Many consumers attended with a focus on learning more about health research to enrich their understanding to inform peer support, research engagement, patient or consumer advocacy and ultimately to create service change. Incorporating these perspectives has the potential to improve the conference experience for consumer delegates and enhance the overall conference focus. Findings confirm that deliberate thought and planning are needed to value the expertise of consumers as health service end users and research partners while acknowledging their potential vulnerability in sharing their healthcare experiences in a conference forum [9]. Respecting the consumer perspective and involvement is reflected in considerations such as logistical planning (e.g., financial reimbursement), as well as in how consumers are approached and included in communication (e.g., limited communication via written English or health literacy considerations) [3, 9]. Similarly, clarity about opportunities for consumer engagement and transparency about consumer roles at conferences is vital [5, 9].

Partnering with consumers throughout all stages of conference planning could facilitate the inclusion of consumer experiences and motivation for attendance to influence conference content, logistics and presentation style. Limited consumer input at a governance level from the inception of conference planning was a gap in the organisational planning and will be addressed for future conferences. A deliberate sharing of power in conference governance is needed to authentically partner with consumers [3, 8]. In future conferences, as in other health service activities, this will be reflected in consumer involvement in committees or groups responsible for planning conference content, scope and logistics. Such power sharing will be evidenced in transparency of the conference purpose, intended audience and the role of consumers in conference processes. Significantly, this shift in focus has the potential to enhance the overall conference quality, content and focus, providing new insights and opportunities that have been widely recognised as inclusive health research.

## 2 Conclusion/Implications

The 2022 Health Services Research Conference provided an opportunity to test various strategies to facilitate consumer and researcher participation and interaction, including scholarships, a buddy system, designated quiet space and consumer co‐chairing of sessions. Our experience reinforces the need to move from consultation with consumers to empowering consumers as members of the multidisciplinary teams conducting and presenting research. Feedback from participants highlighted successes and areas for improvement, indicating the need for clear communication and guidelines, particularly the shared understanding of the role of consumer attendees. These insights suggest an ongoing need for collaborative planning and more structured consumer involvement throughout the conference planning and design cycle.

## Author Contributions

**Bronwyn Newman:** conceptualisation, writing–original draft, writing–review and editing. **Janelle Bowden:** conceptualisation and writing–review, editing. **Rebecca Jessup:** conceptualisation, writing–review and editing. **Lauren J. Christie:** conceptualisation, writing–review and editing. **Ann Livingstone:** conceptualisation, writing–review and editing. **Mitchell Sarkies:** conceptualisation, writing–review and editing. **Anagha Killedar:** conceptualisation, writing–review and editing. **Carole Vleeskens:** conceptualisation, writing–review and editing. **Mashreka Sarwar:** conceptualisation, writing–review and editing. **Thit Tieu:** conceptualisation, writing–review and editing. **Saran Chamberlain:** conceptualisation, writing–review and editing. **Reema Harrison:** conceptualisation, writing–review and editing, writing–original draft. **Alison Pearce:** conceptualisation, project administration, writing–review and editing, writing–original draft.

## Conflicts of Interest

The authors declare no conflicts of interest.

## Data Availability

The data that support the findings of this study are available on request from the corresponding author. The data are not publicly available due to privacy or ethical restrictions.
